# A Within-Individual Examination of the Predictors of Gun Carrying During Adolescence and Young Adulthood Among Young Men

**DOI:** 10.1007/s10964-021-01464-6

**Published:** 2021-07-16

**Authors:** Jordan Beardslee, Emily Kan, Cortney Simmons, Dustin Pardini, Monica Peniche, Paul J. Frick, Laurence Steinberg, Elizabeth Cauffman

**Affiliations:** 1 Department of Psychological Science, University of California, Irvine, CA 92697-7085 USA; 2 Department of Psychology, Yale University, New Haven, USA; 3 School of Criminology and Criminal Justice, Arizona State University, Tempe, USA; 4 Department of Psychology, Louisiana State University, Baton Rouge, USA; 5 Institute for Learning Sciences & Teacher Education, Australian Catholic University, Brisbane, Queensland, Australia; 6 Department of Psychology, Temple University, Philadelphia, USA; 7 King Abdulaziz University, Jeddah, Saudi Arabia

**Keywords:** Gun carrying, Risk factors, Firearm carrying, Adolescent offending

## Abstract

Although prior studies have identified several risk factors for gun carrying, no prior longitudinal studies have examined a comprehensive set of explanatory factors together in within-individual change models or examined whether the predictors of gun carrying change across adolescence and early young adulthood. The present study fills these gaps by examining the predictive utility of several risk factors for gun carrying, and by examining whether any of the associations vary by age. The sample included 1216 young men who were arrested for the first time during adolescence (approximately 15 years old) and interviewed regularly for 5 years (until approximately 20 years old) after the first arrest. The outcome was youth-self-reported gun carrying and the risk factors included several variables consistent with various explanations for gun carrying (psychosocial maturity deficits; antisocial behavioral style; socialization; victimization). Research questions were addressed with fixed effects dynamic panel models (within-individual change models). Results showed that the most robust predictors of gun carrying were increased exposure to guns and gun-related violence and increased engagement in other antisocial and illegal behavior. The results emphasize the specific etiology of gun carrying and the potential social contagion effect of gun-related events. Overall, the study points to the need for prevention and intervention programs to specifically target the reduction of the real and perceived prevalence of gun-related events in young men’s lives.

## Introduction

Adolescents and young adults who carry firearms put themselves and those around them at risk for violent injuries and death (Branas et al., 2009; Carter et al., 2013; Loughran et al., 2016; Pickett et al., 2005). As such, it is critical to identify the factors that place youth most at risk for gun carrying. Although some studies have focused on risk factors that are related to the individual, such as deficits in psychosocial maturity (Lee et al., 2020) or engaging in other antisocial and illegal behaviors (Docherty et al., 2019a, 2019b), other studies suggest that the primary reasons for youth gun carrying are contextual, such as socialization processes (Wilkinson et al., 2009) or self-protection after victimization (Oliphant et al., 2019). In addition, given the many developmental and social differences between adolescents and young adults, it is possible that the predictors of gun carrying change with age. Unfortunately, no prior longitudinal study has examined a comprehensive set of risk factors together, particularly while controlling for youths’ prior propensity to carry a gun, or examined whether the salience of the risk factors for gun carrying changes across adolescence and the transition to young adulthood. The present study addresses these gaps by simultaneously examining the associations between several potential explanatory factors and gun carrying, and by examining whether the magnitude of these associations changed with age.

### Prevalence and Seriousness of Gun Violence and Gun Carrying in the United States

Gun violence is a serious public health issue in the United States. During the recent decade (2010–2019), over 350,000 people in the United States died as a result of gun violence, which resulted in over 8 million years of life lost (Centers for Disease Control, 2021b). It is now well documented that gun violence disproportionately impacts young men (Oliphant et al., 2019). Males account for approximately 85% of all deaths related to gun violence (Centers for Disease Control, 2021a), and in 2019, over 3800 15-to-24-year-old men were killed by guns (excluding suicide and unintentional death; Centers for Disease Control, 2021b). Moreover, more than 71% of these victims were young men of color (Centers for Disease Control, 2021b). Indeed, firearm homicide was the leading cause of death for 15-to-24-year-old Black males in 2019 (Centers for Disease Control, 2021b).

Gun carrying, a common precursor to gun violence (Pardini et al., 2020), is also fairly common in the United States. Approximately 5–10% of community youth report carrying a gun in the past year (Centers for Disease Control, 2012; Oliphant et al., 2019) and approximately 20% of (somewhat high risk) adolescents report carrying a gun during adolescence (Beardslee et al., 2019). One study of adolescents convicted of serious offenses found that almost half (46%) of the sample carried a gun during the 7-year study period and almost a quarter (23%) engaged in gun violence during this time period (Pardini et al., 2020). Another study with detained youth found that approximately 73% had used a firearm prior to age 18 (Teplin, 2019). Given the high prevalence and catastrophic consequences associated with gun violence, particularly for young men of color, it is critical to identify the risk factors that may lead young men to carry (and potentially use) guns.

### Potential Explanations for Gun Carrying During Adolescence and Young Adulthood

Researchers studying the risk factors for gun carrying generally focus on characteristics about the individual (e.g., history of aggression/illegal activity; impulsivity) or the context to which the individual is exposed (e.g., victimization; peer delinquency; for reviews, see Oliphant et al., 2019; Schmidt et al., 2019). Studies examining developmental change in gun carrying generally find that gun carrying tends to be less stable and more episodic than other illegal behavior (Dong & Wiebe, 2018; Steinman & Zimmerman, 2003), and that the prevalence of gun carrying and gun violence generally peak during the early 20 s (Beardslee et al., 2018; Pardini et al., 2020). As such, it is critical to identify the time-varying within-individual risk factors that explain why an adolescent or young adult may carry a gun in some years but not others.

#### Deficits in psychosocial maturity

Youth may carry guns because of diminished psychosocial maturity, which encompasses several aspects of development (e.g., impulse control; future orientation) that influence adolescent decision-making (Greenberger & Sørensen, 1974; Steinberg & Cauffman, 1996). An adolescent boy who is unable to consider the long-term consequences of his actions or control antisocial impulses may be more likely to carry a gun because he is not thinking (or does not care) about the potential danger he could inflict or the legal troubles he could encounter. While many prior studies have found significant associations between components of psychosocial maturity (e.g., impulsivity) and serious offending in general (Bechtold et al., 2014; Fine et al., 2016; Monahan et al., 2013), only a few longitudinal studies have looked at psychosocial maturity specifically in relation to firearm usage (see Lee et al., 2020; Pardini et al., 2020; Rowan et al., 2019). For example, one study with a relatively high-risk community sample found that future orientation may mediate the between-person associations between exposure to violence and subsequent gun carrying (Lee et al. 2020). Furthermore, a study of serious adolescent offenders found that low future orientation was a robust predictor of gun violence in between-individual models but not within-individual models, suggesting that selection effects or omitted confounding variables may have accounted for the between-individual effects (Rowan et al. 2019). As such, it is not clear whether changes in psychosocial maturity would relate to within-individual changes in gun carrying.

#### Antisocial behavioral style

A broad preference for engaging in antisocial behavior, which may include the tendency to engage in a variety of antisocial and illegal behaviors, may increase the likelihood that youth carry guns. Indeed, prior work suggests that the majority of offenders—especially young offenders—do not specialize in certain types of illegal behavior (Piquero, 2000; Simon, 1997). This suggests that engaging in one type of illegal behavior, such as theft, may lead to (or facilitate) another, such as gun carrying. For example, prior studies of justice-system-involved and community youth have consistently found that drug dealing (Docherty et al., 2019a, 2019b; Lizotte et al., 2000; Vaughn et al., 2012), theft/property offending (Vaughn et al., 2019), aggressive/violent offending (Docherty et al., 2019a; Spano, 2012), and conduct/externalizing problems (Loeber et al., 2004; Vaughn et al., 2016) are significantly related to gun carrying and gun violence. For example, one study with adolescents convicted of serious offenses used within-individual change models with 84 consecutive months of data and found a strong and consistent association between drug dealing and gun carrying (Docherty et al., 2019b). Specifically, the study found that gun carrying increased slightly before a drug dealing episode, increased dramatically during the month of drug dealing, and declined rapidly after the drug dealing episode ended but remained higher than initial levels (Docherty et al., 2019b). Although the robust within-person association between drug dealing and gun carrying was compelling in this study, this study did not include measures of psychosocial maturity, parental gun carrying, parent non-gun carrying, or prior gun carrying, and this study was focused on understanding the predictors of gun carrying in a relatively high risk sample of serious adolescent offenders.

Nonetheless, adolescents may be more likely to carry guns during years when they engage in other types of offending because guns may facilitate or support other antisocial goals and activities. Because most of the longitudinal studies in this area tested the antisocial behavior explanation with between-individual models, the extent to which these factors can be used to understand within-individual fluctuations in gun carrying is less understood.

#### Social influence or contagion effect

Exposure to delinquent or gun carrying peers and parents may also increase youths’ odds of gun carrying. Consistent with a socialization or social influence hypothesis, youth may be directly and indirectly taught or encouraged to carry guns by close members of their social network (i.e., peers, parents). Indeed, several studies found that having peers who carry guns (Cao et al., 2008; Hemenway et al., 2011; Lizotte et al., 2000; Robertson et al., 2020) is a significant risk factor for gun carrying and other violence. One study of adolescent and young adults convicted of violent offenses, 92% of whom reported having a gun, found that over 95% of participants reported having peers who carried guns, and over 78% had peers who used guns to commit crimes (Wilkinson et al., 2009).

In addition, significant associations between general and non-gun peer delinquency with gun carrying (e.g., Keil et al., 2020) suggest that antisocial peers may contribute to gun carrying by potentially increasing access to illegal guns or by encouraging antisocial behaviors in general— even if they do not specifically carry guns themselves. In addition to peers, parents are also important socializing agents. Although researchers have examined a variety of parenting factors in relation to youth antisocial behavior and offending (Barnes et al., 2006; Guo et al., 2002; Johnson et al., 2011; Van Ryzin et al., 2012), the extent to which an adolescent or young adult’s parent’s gun carrying and general offending are associated with his own gun carrying is unclear. However, results of prior studies indicate that having “guns in the home” is a significant risk factor for youth gun carrying (e.g., Molnar et al., 2004), suggesting that parent gun carrying may be an important factor for understanding youth gun carrying.

#### Victimization

One unfortunate reality is that many youth in the United States, particularly justice-system-involved youth, are exposed to serious violence. In fact, one study estimated that almost 10% of justice-system-involved male youth in Chicago were injured by a gun prior to age 18 and nearly 33% of the sample had been injured or killed by a firearm prior to approximately age 32 (Teplin, 2019). Another study that followed youth after their first contact with the justice system found that nearly 39% of youth were shot or saw someone else get shot during the 5 years after their first arrest (Shulman et al., 2021). Similar to justice-system-involved samples, youth in low income neighborhoods are also at an elevated risk of being exposed to gun violence. One study found that approximately 16% of adolescent and young adults in a lower income neighborhood in Connecticut witnessed someone being shot in their lifetime (Santilli et al., 2017).

One possible reason that adolescents and young adults may carry guns is for self-protection or retaliation (Oliphant et al., 2019). In line with this explanation, youth who experience or witness gun and/or non-gun violence are potentially at risk for gun carrying because of a desire to protect themselves from future attacks or because of a desire to retaliate against their attackers (Kleck & Gertz, 1998; Sheley & Wright, 1993; Spano & Bolland, 2013; Spano et al., 2010). One published review on the predictors of gun carrying supported this hypothesis by finding that retrospective reports of a desire for self-protection was one of the most consistent predictors of gun carrying (see Oliphant et al., 2019). Additional support for the self-protection/retaliation hypothesis has been found in a variety of studies with justice-system-involved and community youth. For example, prior studies with within-person and between-person models have found that exposure to gun violence (Beardslee et al., 2019; Sumner et al., 2016), exposure to non-gun violence (Rowan et al., 2019), and exposure to general violence (Pardini et al., 2020; Reid et al., 2017; Spano, 2012; Spano & Bolland, 2013) are significantly related to gun carrying and gun violence among adolescents and young adults.

#### Demographic factors related to gun carrying

As mentioned throughout the introduction, there are demographic factors that have been associated with gun carrying. One such factor is age: gun carrying tends to gradually increase throughout adolescence and early young adulthood, often peaking during the early 20 s. Furthermore, gun carrying and gun violence are both disproportionately higher among males and among justice-system-involved youth (Oliphant et al., 2019). Many studies also find that gun violence is concentrated in urban, metropolitan, and lower socioeconomic neighborhoods (Oliphant et al., 2019). Although prior workst suggests race differences in the prevalance of gun carrying, conflicting findings have been reported based on geography (i.e., urban versus rural; Oliphant et al., 2019).

### Limitations In Prior Work

Although prior studies identified several important risk factors for gun carrying, there are many limitations in the existing body of work. First, no prior study simultaneously examined all of the potential explanatory while also controlling for the youths’ prior gun carrying. Of the existing longitudinal studies in this area, most studies focused on specific risk factors such as exposure to violence (Beardslee et al., 2018; Spano & Bollard, 2013), drug dealing (Docherty et al., [Bibr CR17][Bibr CR17]; Lizotte et al., 2000), peer delinquency (Docherty et al., 2019a; Lizotte et al., 2000), conduct problems/aggressive behavior (Docherty et al., 2019a; Spano, 2012), or neighborhood/socio-economic status (Beardslee et al., 2021; Docherty et al., 2019a). None of these studies controlled for all the other relevant behavioral, psychosocial, and contextual factors. While one study included prior gun carrying as a covariate, this study utilized between-individual models to examine perpetrated gun violence and did not control for future orientation, exposure to gun violence, or parental gun carrying and non-gun offending (Pardini et al., 2020). Another longitudinal study with the same sample of serious adolescent offenders as Pardini and colleagues (2020) examined the predictors of gun violence (not gun carrying), but did not control for prior gun carrying, exposure to gun violence, peer gun carrying, impulse control, or parental gun carrying and non-gun offending (Rowan et al., 2019).

Additionally, it is unknown whether the predictors of gun carrying change with age. Given that prior studies have found age differences in the predictors of other types of antisocial behavior (Fergusson et al., 2002; Monahan et al., 2009), it is important to examine whether the nature of the risk factors for gun carrying changes with age. Indeed, there are many cognitive, socio-emotional, and contextual differences between adolescents and young adults, and these differences may impact the degree to which risk factors are related to gun carrying. For example, developmental immaturities during adolescence may render adolescents more susceptible to adverse contextual experiences than adults. In addition, the most salient source of social influence may change across development. While parents’ behavior may have greater influence during early adolescence, peers may be more influential in late adolescence and early young adulthood. There is some evidence of potential age differences in the predictors of gun carrying, but existing studies are limited by their comparison of juveniles to adults (Decker et al., 1997; Watkins et al., 2008) or their utilization of between-individual statistical models and a limited set of explanatory factors (Lizotte et al., 2000).

## The Current Study

The present study was designed to overcome the limitations in prior work by testing whether several psychological, behavioral, and contextual risk factors were associated with gun carrying during adolescence and early young adulthood. Risk factors in the four primary domains (psychosocial maturity, behavioral, social influence, victimization) were examined simultaneously to identify the strongest predictors of gun carrying. Analyses also controlled for other potential explanatory factors and youths’ prior propensity to carry a gun. The present study was also able to examine the main effects of relevant time-stable demographic factors, such as age at first arrest (i.e., time 1) and race/ethnicity. It is important to examine all factors together given that many of the factors are likely related (i.e., individuals with a proclivity for antisocial behaviors may also associate with like-minded peers). Considering the many differences between adolescence and young adulthood, the present study also examined whether the nature of the associations between the risk factors and gun carrying changed with age.

Given that gun violence is disproportionately higher among males and justice-system-involved youth (Oliphant et al., 2019), the research questions were addressed using data drawn from a 5-year longitudinal study of young men who had a history of law-breaking behavior and were housed predominately within the community. The moderately high-risk nature of the sample ensured that there was sufficient within-person variability in gun carrying across the study period, which allowed the use of fixed effects dynamic panel models to estimate within-individual change in the likelihood of gun carrying. Within-individual change models are advantageous because they treat individuals as their own control variables, which means that all unchanging factors about individuals and their environments are automatically controlled (Allison, 2009). As such, these models reduce the potential impact of selection effects, shared risk factors, and confounding variables, and offer stronger tests of potential causal pathways than traditional between-individual models. By focusing exclusively on within-individual change, the analysis sought to understand why a person carries a gun in some years but not others. This is a fundamentally different question than one that asks why one person carries a gun at all, but another person never does—which is typically the question that can be answered with traditional between-individual models (which is an interesting question but can be confounded by the many differences between the types of people who do and do not carry guns).

## Methods

### Participants

The sample included the 1216 male youth enrolled in the Crossroads Study (http://sites.uci.edu/crossroadsinfo/; see Cauffman et al., 2021). Youth were eligible to participate in the Crossroads Study if they were recently arrested for the first time for a low or moderate offense (e.g., vandalism; theft; mostly misdemeanors), were between 13 and 17 years old, and were being processed in Philadelphia, Pennsylvania (*N* = 533); Orange County, California (*N* = 532); or Jefferson Parish, Louisiana (*N* = 151). The study investigators selected these three locales to enhance the diversity and representativeness of the sample. The combined sample was racially and ethnically diverse: 46% Latinx/Hispanic, 37% Black/African American, 15% White, and 2% self-identified as multi-racial, multi-ethnic, or another race or ethnicity. Of those who were contacted, approximately 72% agreed to participate in the study.

### Procedures

Researchers identified potential study participants through a collaborative process with the probation departments and courts in each site. From 2011 to 2013, courts and/or probation departments provided research staff with a list of juveniles who were arrested in the past week and research staff determined whether each juvenile met the criteria for study inclusion (e.g., eligible charge; within age range; no prior history of arrests). After researchers identified the eligible youth, research staff contacted youth and their parents via the telephone, email, or a house visit within six weeks of case dispositions. All contact information was obtained from the probation department, court records, and/or publicly accessible information sources.

Eligible youth and their families were informed of the nature of the study and invited to participate. Youth who were interested in the study underwent an extensive parent-consent and youth-assent process, which included a detailed description of the study along with a description of the Department of Justice’s Privacy Certificate. The advantage of the Privacy Certificate was that all data obtained as part of the Crossroads study were protected against subpoenas, court orders, and/or other involuntary disclosures. The only exceptions to the promise of confidentiality were instances where the participant revealed suicidal thoughts, homicidal thoughts, or serious plans to engage in future criminal conduct.

Project staff interviewed participants using a secure, computer-assisted program. Interviews were about 2 to 3 h and were conducted in the community or wherever the youth was housed (e.g., detention center; jail). Phone interviews were conducted when participants were not physically accessible (e.g., moved out of state). Youth completed their first interview (i.e., “baseline”; time 1) within six weeks of receiving the disposition for the charges associated with their first arrest. After the baseline interview, Crossroads research staff members interviewed participants biannually for three years and then annually for two years (baseline plus biannual/annual interviews over a 5-year period; 9 total measurement occasions). Youth were between 13 and 17 years of age (*M*_age_ = 15.29) at baseline and between 18 and 23 years of age (*M*_age_ = 20.29) at the 5-year follow up interview. Slightly more than half of all interviews were conducted in participants’ homes (52%). The remaining interviews were conducted in coffee shops or somewhere in the community (36%), on the phone (6%), in detention, jail, or other locked facilities (5%), or in participants’ friends’ or family members’ homes (1%). Interviews assessed a variety of domains, including participants’ thoughts, behaviors, attitudes, and contextual experiences. The variables used in the present analysis were self-reported by the youth at all 9 interviews. Retention across the 5-year study was high: between 85% and 95% of the initial sample completed each of the follow up interviews (missing data discussed below).

The research team utilized many strategies to prevent attrition. For example, at the end of each interview, participants were asked to provide all available contact information for themselves and for friends and family members who might know how to contact the youth in the future. Additionally, the research team was flexible when scheduling the interviews. Interviewers were able to meet participants wherever and whenever was most convenient for the participant. Youth were also financially compensated for their time according to an escalating payment plan. The Institutional Review Board at the participating universities approved all recruitment and study procedures. More detailed information about the Crossroads study has been published previously (Cauffman et al., 2021).

### Measures

#### Gun carrying

At each interview, one item from the Self-Report of Offending scale (SRO; Huizinga et al., 1991) was utilized to assess whether the participant had carried a gun since the previous interview (1 = yes, carried a gun; 0 = no, did not carry a gun). It was assumed that participants who reported shooting another person during the recall period had also carried a gun. Gun carrying was re-coded to “missing” for periods during which the participant was housed in a secure, locked facility (e.g., juvenile hall or local jail) for the entire recall period because it is unknown whether a participant would have carried a gun in that recall period given that the facility would have prohibited access to one (*<*2% of interviews).

#### Psychosocial maturity

##### Impulse control

A measure of impulse control was derived from the Impulse Control scale from the Weinberger Adjustment Inventory (Weinberger & Schwartz, 1990). The Impulse Control scale consisted of 8 items that measured the extent to which the participant was generally able to inhibit impulsive behavior. Youth responded to each item using a 5-point Likert scale that ranged from 1 (“false”) to 5 (“true”). Sample items include behaviors such as, “I do things without giving them enough thought” (reverse scored) and “I say the first thing that comes into my mind without thinking enough about it” (reverse scored). A total impulse control was created by calculating the mean of the 8 items, with higher scores indicative of a greater ability to control impulsive behavior. Internal consistency for impulse control was acceptable: mean *α* = 0.770, range *α* = 0.741 to 0.790.

##### Future orientation

A measure of future orientation was obtained using 15 items from the Future Outlook Inventory (Cauffman & Woolard, 1999, unpublished manuscript). Each item asked youth to state the degree to which a statement reflected their beliefs about the future. Sample items include “I will keep working at difficult, boring tasks if I know they will help me get ahead later” and “I think about how things might be in the future.” Youth responded to each item using a 4-point Likert scale that ranged from 1 (“never true”) to 4 (“always true”). A total measure of future orientation was calculated by summing the 15 items, with higher scores indicating a greater orientation toward the future. Internal consistency for future orientation was acceptable: mean *α* = 0.712, range *α* = 0.657 to 0.743.

#### Behavioral

##### Prior gun carrying

As described previously, one item from the SRO (Huizinga et al., 1991) was used to measure whether the participant carried a gun during the recall period. For the primary analysis, gun carrying during the prior year (Time–1) was used as a time-varying covariate.

##### Non-gun theft and property offending

Nine items from the SRO were used to measure non-gun related theft and property offending, which included behaviors such as vandalism, shoplifting, and joyriding. For each item, participants were asked whether they had engaged in the behavior during the recall period. The nine items were combined to create a single binary measure of theft and property offending (1 = yes engaged in at least one theft or property offense; 0 = did not engage in any theft or property offending). Consistent with gun carrying, theft and property offending was set to “missing” for periods during which the participant was housed in a secure, locked facility for the entire recall period.

##### Non-gun aggressive and violent offending

Four items from the SRO were used to measure non-gun related aggressive and violent offending, which included behaviors such as assault, fighting, and robbery. For each item, participants were asked whether they had engaged in the behavior during the recall period. If the participant engaged in any of the four behaviors, the non-gun aggressive and violent offending variable was coded as a “1.” If the participant did not engage in any non-gun aggressive and violent offending, the participant received a score of “0” for this variable. Consistent with gun carrying, aggressive and violent offending was set to “missing” for periods during which the participant was housed in a secure, locked facility for the entire recall period.

##### Drug dealing

Drug dealing was measured with 2 items from the SRO scale (Huizinga et al., 1991). At each interview, researchers asked youth whether they had sold marijuana and whether they had sold other illicit drugs during the recall period. The 2 items were combined to create a single measure of drug dealing (1 = any drug dealing; 0 = no drug dealing) at each time-point.

#### Social influence

##### Peer gun carrying

Peer gun carrying was measured with a single item from the Association with Deviant Peers scale (Thornberry et al., 1994). The item asked youth to state the proportion of friends who had carried a gun during the recall period. Youth responded to the item using a 5-point Likert scale that ranged from 1 (“none of them”) to 5 (“all of them”).

##### Peer general (non-gun) offending

Peer general offending (exclusive of gun use items) was based on a subset of items from the Association with Deviant Peers scale (Thornberry et al., 1994). At each interview, 8 items asked the participant to state the proportion of friends who had engaged in different delinquent and antisocial activities during the recall period. Youth responded to each item using a 5-point Likert scale that ranged from 1 (“none of them”) to 5 (“all of them”). Sample behaviors include property damage, physical violence/fighting, burglary/theft, and drug dealing. A total peer general offending scale was created by calculating the mean of the 8 items, with higher scores indicative of greater exposure to delinquent peers. Internal consistency for peer general offending was high: mean *α* = 0.863, range = 0.848 to 0.881.

##### Parent gun carrying

Parent gun carrying was measured at each time-point with a single item from a modified version of the Association with Deviant Peers scale (Thornberry et al., 1994). Specifically, a single item measured whether either of the youth’s parents had carried a gun during the recall period (1 = paternal or maternal guardian carried a gun; 0 = neither parent carried a gun).

##### Parent general (non-gun) offending

The research team measured parent general offending (exclusive of gun items) with 8 items from a modified version of the Association with Deviant Peers scale (Thornberry et al., 1994). For each item that was used to measure peer general offending, a parallel item asked whether either of the youth’s parents had engaged in the behavior during the recall period (1 = one or more parent engaged in behavior; 0 = neither parent engaged in behavior). Similar to peer general offending, sample behaviors include property damage, physical violence/fighting, burglary/theft, and drug dealing. The final parent general offending variable was a binary variable that indicated whether the participants’ parents engaged in any of the general non-gun offending items during the recall period (1 = paternal or maternal guardian engaged in at least one general non-gun offense; 0 = neither parent engaged in non-gun offending).

#### Victimization

##### Exposure to gun violence

The research team used four items from the Exposure to Violence (ETV) inventory (Selner-O’Hagan et al., 1998) to measure exposure to gun violence during each recall period. At each interview, items measured whether the participant was shot (i.e., shot and hit) or shot at (i.e., shot at but bullet missed), and whether he witnessed someone else get shot or shot at. The four items were combined to create a single binary variable indexing whether the participant was the victim or witness of gun-related violence during each recall period (1 = witness/victim of gun violence; 0 = did not experience or witness gun violence).

##### Exposure to general (non-gun) violence

Six items from the ETV inventory (Selner-O’Hagan et al., 1998) were used to measure the degree to which the participant was exposed to non-gun violence during each recall period. At each interview, the participant reported whether he experienced or witnessed three specific (non-gun) violent events. Sample non-gun violent events include whether the participant was chased/beaten up/attacked and whether the participant witnessed someone else getting chased/beaten up/attacked. A dichotomous variable was created to index whether participants experienced any of the general (non-gun) violent events during each recall period (1 = victim or witness of general violence; 0 = did not experience or witness any general violence).

#### Time-Stable Demographic Factors

##### Age

Youth’s age at baseline/time 1 was calculated based on the date of the interview and the youth’s date of birth.

##### Race and ethnicity

Participants reported their race and ethnicity at the first interview. Race/ethnicity was coded into a 4-categories: White, Black, Hispanic, and Bi-racial/Other.

##### Parent highest education

Parent highest education was used as a proxy for socioeconomic status. Youth reported their parent’s highest education by using a 10-point scale that ranged from some grade school to professional or graduate degree.

##### IQ proxy

A proxy for IQ was created with the vocabulary and matrix reasoning subscales of the Wechsler Abbreviated Scale of Intelligence (Wechsler, 1999).

### Plan of Analysis

Research questions were addressed with dynamic panel models in fixed effects binary logistic regressions within a structural equation framework (Bollen & Brand, 2010; Williams et al., 2018). These models are ideal for the current study because they can be used to understand within-individual change in an outcome variable, which controls for all time-stable effects of time-stable confounding variables. These models are also advantageous compared to traditional fixed effects models because it is possible to obtain (random effect) estimates for time-stable variables (such as race or age at baseline) and control for prior levels of the dependent variable. The general specification for the time-varying, within-individual change component of the primary model was (Allison, 2009):

GunCarrying_it_ = β_1_ImpulseControl_it_ + β_2_FutureOrientation_it_ + β_3_PriorGunCarrying_i,t-1_ + β_4_NonGunTheftProp_it_ + β_5_NonGunAggViolence_it_ + β_6_DrugDealing_it_ + β_7_PeerGunCarry_it_ + β_8_PeerNonGunOffending_it_ + β_9_ParentGunCarry_it_ + β_10_ParentNonGunOffending_it_ + β_11_ETVGun_it_ + β_12_ETVNonGun_it_ + γ Demographics_i_ + *α*_i_ + µ_t_ + ε_it_

Where:

- Gun Carrying_it_ represents the probability of carrying a gun at time t for individual i.
- β_1_- β_12_ represents the association between the risk factor and gun carrying
  - In the primary models, all coefficients (β_1_- β_12_) were constrained to be equal across time. Additional models examined whether relaxing this constraint improved model fit.
- *γ*_i_ represents the combined effect of the time-invariant demographic factors (these are essentially random effects)
- *α*_i_ represents a fixed constant indexing the underlying probability of gun carrying across the time series for each individual i
- µ_t_ represents a fixed constant for each time-point (which accounts for changes in gun carrying over time)
- ε_it_ represent a residual variance for individual i at time t

After the primary models, the authors examined whether the magnitude of any of the associations between the risk factors and gun carrying changed during the study period. Because there is not a straightforward way to examine age interactions in these models, two strategies were utilized in the present study. First, the authors compared the primary model (which had time-invariatn effects of the risk factors) to models where each risk factor was freely estimated across time using log likelihood chi-square tests. A constrained and freely estimated model was compared for each risk factor, while controlling for all other risk factors from the primary model. In these models, a significant log likelihood chi-square test revealed that the unconstrained model may be a better fit to the data than a constrained model. Because these models may capitalize on chance when identifying fluctuations in parameter estimates over time, the authors also estimated interactions between age at time 1 and all of the primary risk factors.

As a supplemental analysis, the primary model was repeated with the three non-gun antisocial behavior and offending items as the outcomes. These analyses were conducted to evaluate whether the predictors of gun carrying were similar to the predictors of other types of antisocial behavior and offending. In particular, these models were used to predict non-gun theft and property offending, non-gun aggressive and violent offending, and drug dealing. All risk factors used in the primary models were used to predict the non-gun offending items. Gun carrying was also used as a predictor. The supplemental models also included the lagged dependent variable (consistent with the primary models).

Finally, two sensitivity analyses were conducted. In the first model, the three variables that were dichotomized in the primary analysis (non-gun theft and property offending, non-gun aggressive and violent offending, and parent non-gun offending) were left in their original forms (variety score counts). In the second model, the primary analysis was conducted without the imputed datasets (imputed data discussed below). All models in the present study were estimated with maximum likelihood estimation and conducted in Mplus version 8 with 25 imputed datasets (discussed below).

### Missing Data

Crossroads study participants completed over 92% of all possible interviews (9 total interviews X 1216 participants = 10,944 possible interviews), resulting in over 10,000 data points for possible inclusion in the present study. Of the missing interviews (approximately 8% of all interviews), the primary reason for missing data was because the research team was unable to contact or locate the participant during the search window (41.7% of *missed* interviews). Only 24% of *missed* interviews were because the participant withdrew from the study, 10% of *missed* interviews were due to chronic no-shows which resulted in an expired search window, and 9% of *missed* interviews were because a legal actor (e.g., lawyer, justice system facility staff) prevented us from contacting/accessing the participant, and the rest were missed due to other reasons (e.g., military deployment, death, hospitalization).

Of the 9 possible interviews for each participant, 71% of the participants missed none of the interviews, 15% missed one interview, 5% missed two interviews, 3% missed three interviews, and 6% missed four or more interviews. The authors examined whether having missing data was associated with any of the study variables measured at baseline using binary logistic regressions. These analyses revealed that having missing data was slightly more likely among Black than White (*p* = 0.024) and Hispanic (*p* < 0.001) youth, among those with lower IQs (*p* = 0.006), among those who had carried a gun prior to baseline (*p* = 0.002), and among boys who had peers who carried guns (*p* = 0.007). However, having missing data was not associated with parent highest education (*p* = 0.544), age at time 1 (*p* = 0.070), impulse control (*p* = 0.385), future orientation (*p* = 0.708), non-gun theft & property offending (*p* = 0.445), non-gun aggressive & violent offending (*p* = 0.355), drug dealing (*p* = 0.231), peer non-gun delinquency (*p* = 0.065), parent gun carrying (*p* = 0.879), parent non-gun delinquency (*p* = 0.161), gun victimization (*p* = 0.139), and non-gun victimization (*p* = 0.156). Thus, it is unlikely that missing data had a substantive impact on the results produced from the analyses conducted in the present study. Furthermore, experts in this area have noted that missing data due to participant attrition is less problematic in within-individual models than between-individual models (Hill et al. 2017). Nonetheless, in order to prevent cases with missing data on the predictor variables from being dropped from the analysis, 25 data sets were imputed, and analyses were conducted with the imputed datasets. All results were combined using Rubin’s rules (Rubin, 1987).

## Results

### Descriptive Statistics for Gun-Related Study Variables

**Fig. 1:**
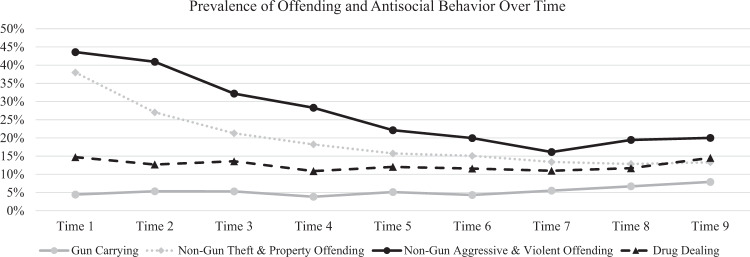
Descriptive statistics for offending and antisocial behavior by time

As demonstrated in Table 1 and Fig. 1, the prevalence of gun carrying gradually increased throughout the study period. Descriptive univariate growth curve models demonstrated that a quadratic model was a better fit to the data than a linear only model (X^2^ for difference testing = 15.41, *p* = 0.004). In the quadratic growth model, the mean and variance were significantly different from zero for the linear slope (mean = 0.83; *p* = 0.050; variance = 0.58, *p* = 0.022) and the mean was significantly different than zero for the quadratic slope (mean = −0.41, *p* = 0.018; variance = 0.07, *p* = 0.199). Taken together, these growth factors suggest that the overall prevalence of gun carrying gradually increased throughout the study period (with diminished growth towards the end of the study period), and that individuals varied in their rate of change in gun carrying over time. See Table 1, Table 2, and Fig. 1 for more descriptive information about the study variables.

**Table 1:** Descriptive statistics for time-varying study variables by time

Study Variable	Time 1 *M* (*SD*)/%	Time 2 *M* (*SD*)/%	Time 3 *M* (*SD*)/%	Time 4 *M* (*SD*)/%	Time 5 *M* (*SD*)/%	Time 6 *M* (*SD*)/%	Time 7 *M* (*SD*)/%	Time 8 *M* (*SD*)/%	Time 9 *M* (*SD*)/%
Gun carrying	4.40%	5.40%	5.30%	3.80%	5.10%	4.30%	5.50%	6.70%	7.90%
Psychosocial maturity predictors									
Impulse control	3.25 (0.86)	3.35 (0.86)	3.31 (0.89)	3.34 (0.89)	3.39 (0.87)	3.38 (0.86)	3.38 (0.87)	3.37 (0.87)	3.44 (0.86)
Future orientation	2.54 (0.52)	2.60 (0.53)	2.67 (0.53)	2.72 (0.53)	2.79 (0.56)	2.83 (0.55)	2.87 (0.54)	2.92 (0.55)	2.98 (0.54)
Behavioral predictors									
Non-gun theft and property offending	37.99%	27.03%	21.28%	18.22%	15.74%	15.09%	13.41%	12.84%	13.31%
Non-gun aggressive and violent offending	43.59%	40.93%	32.19%	28.30%	22.14%	19.96%	16.14%	19.46%	20.02%
Drug dealing	14.72%	12.7%	13.58%	10.89%	12.05%	11.59%	10.98%	11.69%	14.52%
Social influence predictors									
Peer gun carrying	1.26 (0.69)	1.32 (0.81)	1.30 (0.76)	1.31 (0.77)	1.31 (0.77)	1.31 (0.77)	1.32 (0.80)	1.36 (0.84)	1.42 (0.85)
Peer general (non-gun) offending	1.71 (0.67)	1.59 (0.66)	1.55 (0.69)	1.48 (0.63)	1.42 (0.60)	1.39 (0.59)	1.36 (0.57)	1.37 (0.57)	1.36 (0.55)
Parent gun carrying	4.13%	3.74%	2.86%	2.87%	3.25%	2.10%	3.40%	2.98%	3.36%
Parent general (non-gun) offending	17.71%	10.86%	10.28%	7.80%	8.04%	6.20%	5.57%	7.65%	6.62%
Victimization predictors									
Exposure to gun violence	11.68%	12.89%	10.78%	9.83%	8.15%	7.40%	8.34%	10.92%	10.23%
Exposure to general (non-gun) violence	41.94%	41.75%	33.48%	27.83%	24.89%	20.79%	21.85%	20.23%	19.59%

Descriptive statistics are based on observed data (non-imputed). The first 7 interviews (Time 1-Time 7) were conducted in biannual intervals and the last two interviews were conducted annually (Time 8 and Time 9). Mean ages (and SD) at each time-point were: 15.8 (1.3) years at Time 1; 16.3 (1.3) at Time 2; 16.8 (1.3) years at Time 3; 17.3 (1.3) years at Time 4; 17.8 (1.3) years at Time 5; 18.3 (1.3) years at Time 6; 18.8 (1.3) years at Time 7; 19.8 (1.3) years at Time 8; 20.8 (1.3) years at Time 9

**Table 2:** Descriptive statistics for time-invariant demographic predictors

Study variable	*M* (*SD*)	%
Race & Ethnicity		
White		14.8%
Black		36.9%
Hispanic		45.8%
Other		2.5%
Parent highest education (continuous)	5.33 (2.14)	
Parent highest education		
Some grade school		3.5%
Finished grade school		3.9%
Some high school		19.6%
GED		2.2%
High school diploma		32.5%
Business or trade school		2.7%
Some college or graduate of 2 year college		17.5%
College Graduate of four year college		13.4%
Some graduate or professional school beyond college		1.5%
Professional or graduate degree		3.3%
IQ proxy	88.43 (11.59)	
Age (continuous)	15.80 (1.28)	
Age		
13 Years Old		11.2%
14 Years Old		17.3%
15 Years Old		24.7%
16 Years Old		25.5%
17 Years Old		21.3%

Descriptive statistics are based on observed data (non-imputed)

### Predictors of Gun Carrying

**Fig. 2:**
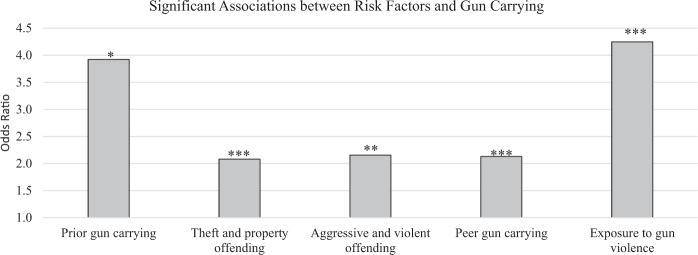
Within-individual associations between the risk factors and gun carrying. Only risk factors that were significantly related to gun carrying are shown in the figure. *p < 0.05, **p < 0.01, ***p < 0.001

When the risk factors from all domains (psychosocial maturity, antisocial behavior, social influence, victimization) were included in the same model (see Table 3, Model 5), the risk factors significantly related to gun carrying were prior gun carrying (*OR* = 3.92, *p* = 0.031), non-gun theft and property offending (*OR* = 2.08, *p* < 0.001), non-gun aggression and violence (*OR* = 2.15, *p* = 0.004), peer gun carrying (*OR* = 2.13, *p* < 0.001), and exposure to gun violence (*OR* = 4.25, *p* < 0.001). As such, young men were significantly more likely to carry guns in years when they engaged in other types of offending (e.g., non-gun theft/property or non-gun aggression/violence) and when they were exposed to guns and gun-related violence (e.g., peer gun carrying, exposure to gun violence)—even after controlling for prior gun carrying and several time-varying confounding factors (see Fig. 2). Interestingly, the coefficients for drug dealing and parent gun carrying were also nearly significant (*OR* = 1.51, *p* = 0.055; *OR* = 1.82, *p* = 0.060, respectively), suggesting that adolescent and young adult men may also carry guns during years when they sell drugs and/or have parents who carry guns. See Table 3 for estimates for all predictors in the fully adjusted model.

**Table 3:** Associations between the time-varying risk factors and time-invariant demographic factors with gun carrying

Predictor	Model 1			Model 2			Model 3			Model 4			Model 5		
	*B*	*SE*	*p*	*B*	*SE*	*p*	*B*	*SE*	*p*	*B*	*SE*	*p*	*B*	*SE*	*p*
Psychosocial Maturity															
Impulse control	**−0.38**	**0.10**	**<0.001**										**−**0.06	0.17	0.738
Future orientation	**−**0.24	0.15	0.107										**−**0.23	0.22	0.295
Behavioral															
Prior gun carrying				**1.04**	**0.20**	**<0.001**							**1.37**	**0.63**	**0.031**
Non-gun theft & property offending				**1.24**	**0.17**	**<0.001**							**0.73**	**0.18**	**<0.001**
Non-gun aggressive & violent offending				**1.02**	**0.16**	**<0.001**							**0.77**	**0.27**	**0.004**
Drug dealing				**0.53**	**0.18**	**0.003**							*0.41*	*0.22*	*0.055*
Social influence															
Peer gun carrying							**0.78**	**0.09**	**<0.001**				**0.76**	**0.09**	**<0.001**
Peer general (non-gun) offending							**0.45**	**0.14**	**0.001**				**−**0.02	0.15	0.909
Parent gun carrying							*0.56*	*0.30*	*0.063*				*0.60*	*0.32*	*0.060*
Parent general (non-gun) offending							**0.48**	**0.20**	**0.017**				0.26	0.23	0.253
Victimization															
Exposure to gun violence										**1.84**	**0.18**	**<0.001**	**1.45**	**0.19**	**<0.001**
Exposure to general (non-gun) violence										**0.80**	**0.17**	**<0.001**	0.27	0.18	0.139
Time-stable demographic factors															
Race & Ethnicity															
Black													0.26	0.35	0.461
Hispanic													0.16	0.31	0.608
Other													**−**0.14	0.51	0.790
Parent highest education													0.06	0.04	0.107
IQ proxy													0.00	0.01	0.849
Age													0.09	0.08	0.260

*Notes*. All models were estimated with binary fixed-effects logistic regressions in a structural equation framework with maximum likelihood estimation (dynamic panel models). Missing data were imputed with 25 datasets. All models also controlled for time. All predictor variables were concurrent with the outcome except the time-invariant demographic variables (which were measured at baseline) and the lagged dependent variable (lagged interval = Time–1)

Bold typeface added to emphasize findings that were significant based on *p* < 0.05. Italic text added to emphasize findings that were nearly significant (*p* < 0.065)

### Predictors of Gun Carrying by Time/Age

In the next stage of the analysis, time-related variations in the associations between the risk factors and gun carrying were first examined by comparing the fully adjusted model from the previous step (Table 3, Model 5) to models wherein each risk factor was freely estimated across time. Although four of these model tests were significant based on the log likelihood X^2^ tests, a close examination of the estimates revealed that almost none of the fluctuations across time were clinically meaningful (see Supplemental Table 1). The only potentially meaningful fluctuation was in regard to the impact of prior of gun carrying, which may have had a stronger impact on gun carrying in the beginning of the study. An examination of the coefficients in the free models for the other three significant X^2^ tests suggested that the minor fluctuations by time were likely due to chance (see Supplemental Table 1). In regard to the interactions with age at time 1, only two of the interactions were significant (i.e., theft and property offending and parent non-gun delinquency; see Supplemental Table 2), which suggested that the magnitude of these two risk factors may be stronger for youth who were younger at time 1 (Age X theft and property offending: *B* = −0.26, *p* = 0.023; Age X parent non-gun: *B* = −0.43, *p* = 0.005). Nonetheless, all things considered, the analyses in the present study did not produce consistent and compelling evidence that the nature of the risk factors for gun carrying substantially changed during adolescence and the transition to young adulthood.

### Supplemental Analyses

The next step of the analysis examined whether the predictors of gun carrying were similar to the predictors of other types of offending. The within-individual associations between the risk factors and non-gun theft and property offending, non-gun aggression and violence, and drug dealing are shown in Table 4. As shown in the table, the predictors of non-gun offending were different than the predictors of gun carrying. For example, young men were significantly more likely to engage in theft and property offending and aggression and violence during years when they experienced significant declines in impulse control and future orientation and when they experienced significant increases in peer non-gun offending, parent non-gun offending, and exposure to non-gun violence. None of these risk factors (i.e., impulse control; future orientation; non-gun peer offending; non-gun parent offending; exposure to non-gun violence) were significantly related to gun carrying in the fully adjusted model. Interestingly, the only risk factor significantly related to drug dealing was prior drug dealing (see Table 4, Model 3).

**Table 4:** Associations between time-varying risk factors and time-invariant demographic factors with non-gun theft and property offending, non-gun violence and aggressive offending, and drug dealing

Predictor	Model 1: Predicting non-gun theft and property offending			Model 2: Predicting non-gun aggression and violence			Model 3: Predicting drug dealing		
	*B*	*SE*	*p*	*B*	*SE*	*p*	*B*	*SE*	*p*
Psychosocial maturity									
Impulse control	**−0.31**	**0.07**	**<0**.**001**	**−0.12**	**0.06**	0.**050**	0.04	0.05	0.394
Future orientation	**−0.44**	**0.11**	**<0.001**	**−0.24**	**0.09**	**0.010**	**−**0.03	0.08	0.742
Behavioral									
Prior dependent variable	**0.56**	**0.08**	**<0.001**	**0.32**	**0.08**	**<0.001**	**0.39**	**0.10**	**<0.001**
Theft and property offending				**0.71**	**0.10**	**<0.001**	0.22	0.12	0.061
Non-gun related aggression and violence	**0.73**	**0.11**	**<0.001**				**−**0.01	0.11	0.903
Gun carrying	**0.83**	**0.18**	**<0.001**	**0.58**	**0.17**	**0.001**	0.32	0.19	0.094
Drug dealing	0.16	0.12	0.185	0.09	0.11	0.388			
Social influence									
Peer gun carrying	**−**0.11	0.07	0.089	**−0.17**	**0.06**	**0.005**	**−**0.07	0.07	0.300
Peer general (non-gun) offending	**1.13**	**0.10**	**<0.001**	**1.10**	**0.09**	**<0.001**	0.17	0.09	0.051
Parent gun carrying	0.19	0.25	0.450	**−**0.13	0.23	0.579	**−**0.04	0.26	0.865
Parent general (non-gun) offending	**0.50**	**0.14**	**<0.001**	**0.41**	**0.12**	**0.001**	0.12	0.14	0.396
Victimization									
Exposure to gun violence	**0.61**	**0.14**	**<0.001**	0.19	0.13	0.135	**−**0.11	0.15	0.467
Exposure to general (non-gun) violence	**0.40**	**0.10**	**<0.001**	**0.76**	**0.09**	**<0.001**	0.09	0.10	0.345
Time-stable demographic factors									
Race & Ethnicity									
Black	0.00	0.17	0.999	0.05	0.14	0.709	**−**0.20	0.12	0.081
Hispanic	0.18	0.16	0.259	0.05	0.14	0.708	0.10	0.11	0.389
Other	**−**0.34	0.35	0.327	0.23	0.28	0.415	**−**0.33	0.26	0.208
Parent highest education	0.01	0.03	0.857	0.01	0.02	0.697	0.00	0.02	0.982
IQ proxy	0.00	0.01	0.799	0.00	0.00	0.694	0.01	0.00	0.053
Age	0.06	0.04	0.166	**−0.25**	**0.04**	**0.000**	**−**0.01	0.03	0.775

*Notes*. All models were estimated with binary fixed-effects logistic regressions in a structural equation framework with maximum likelihood estimation (dynamic panel models). Missing data were imputed with 25 datasets. All models also controlled for time. All predictor variables were concurrent with the outcome except the time-invariant demographic variables (which were measured at baseline) and the lagged dependent variable (lagged interval = Time–1). Note that Time 1 was not included in the model estimating drug dealing because of convergence problems in the imputation model

Bold typeface added to table to emphasize findings that were significant based on *p* < 0.05

### Sensitivity Analyses

In the final stage of the analysis, two sensitivity models were examined. The first issue examined was the decision to dichotomize three of the variables in the primary model. The second issue examined was the decision to impute 25 datasets. The results from these models are shown in Supplemental Table 3 and Supplemental Table 4. As shown in the tables, results are very similar to the primary models.

## Discussion

Although prior studies have identified many important risk factors for gun carrying, the existing body of work suffers from several limitations. Most importantly, no prior study has simultaneously examined four potential explanations for gun carrying together in a single dynamic within-individual change model, particularly while controlling for youths’ prior propensity to carry a gun. Furthermore, no studies examined whether the salience of various risk factors for gun carrying changes across adolescence and the transition to adulthood. Indeed, there are many socio-emotional and contextual differences between adolescents and young adults, and these differences may impact the degree to which risk factors are related to gun carrying. The present study was designed to overcome the shortcomings in prior work by examining the extent to which several psychological, behavioral, and contextual explanatory factors were related to gun carrying during adolescence and early young adulthood, and by examining whether any of the associations changed with age. In general, the strongest within-person predictors of gun carrying were engaging in other antisocial and illegal behavior and exposure to gun-related events (e.g., peer gun carrying, exposure to gun violence).

### Behavioral

The present study found that the more robust predictors of gun carrying were engaging in theft and property offending, engaging in aggression and violence, and to a lesser extent, engaging in drug dealing, consistent with prior work. This finding suggests that young men were more likely to carry guns during periods when they engaged in other types of antisocial and illegal behavior. It is possible that guns are used as tools to facilitate other antisocial goals or for protection while engaging in other illegal activities. It was interesting that the only risk factors related to gun carrying after controlling for co-occurring antisocial behavior and prior gun carrying were exposure to gun-related events such as peer gun carrying and exposure to gun violence in their communities.

### Social Influence

Consistent with socialization and social contagion processes (Tracy et al., 2016), the present study found that youth were more likely to carry guns in years when their peers (and to some extent their parents) carried a gun. It is possible that peers (and possibly parents) promoted youth gun carrying through direct encouragement and indirect modeling, or by normalizing and glorifying guns. It is also possible that these factors were correlated with youth gun carrying because they all signal that the individual was exposed to a social culture in which guns were easily accessible, or because associating with antisocial peers provided access to guns and/or illegal gun markets. An additional potential explanation is that youth who carried guns sought out peer groups where guns were used (i.e., selection). The precise mechanisms linking these factors to gun carrying (and the direction of the effects) were not directly examined in the present study, although the present study did statistically control for youths’ prior propensity to carry a gun, suggesting a greater likelihood of socialization rather than selection. Nonetheless, it was striking that having peers who carried guns was significantly related to youth gun carrying, even after controlling for prior carrying, youths’ own co-occurring antisocial and illegal behavior, psychosocial deficits, victimization, and time-stable demographic factors.

### Victimization

In addition to potentially being socialized by peers (and maybe parents), the results suggest that youth may carry guns for self-protection or retaliation. This is consistent with prior studies showing that victims of violence are significantly more likely than others to carry guns and other weapons (Molnar et al., 2004; Reid et al., 2017; Spano, 2012; Spano & Bolland, 2013; Spano & Bolland, 2011; Van Geel et al., 2014; Vaughn et al., 2006; Vaughn et al., 2012; Wallace, 2017). Interestingly, it was found that youth were more likely to carry guns in years when they were exposed to gun violence but not when they were exposed to non-gun-related violence, similar to prior work with serious adolescent offenders (Beardslee et al., 2018). For example, the odds of carrying a gun in the present study were about 4.3 times higher during years when a youth was the victim or witness of gun violence compared to other years, but gun carrying was not significantly elevated during years when the youth was exposed to serious violence that was not accompanied by guns (i.e., beaten up).

These findings highlight the unique and specific association between gun victimization and gun carrying and suggest that youth gun carrying is most likely to occur during years when the youth witnesses someone getting shot—whether they are the victim or not. It is possible that youth carry guns to protect themselves and others from future gun-related attacks or to retaliate against attackers. Indeed, it is possible that youth chose a weapon of similar lethality when trying to prevent future attacks or retaliating. Importantly, the impact of exposure to gun violence was significant even after controlling for the participant’s own prior gun carrying, theft and property offending, and aggressive and violent offending. The present study did not examine whether gun carrying “worked” in terms of preventing future gun attacks, although prior studies suggest that gun and other weapon carrying actually leads to more, not less, violence (Carter et al., 2013; Pickett et al., 2005; Watts, 2019). For example, one study found that the odds of experiencing gun victimization were almost 2.5 times higher for youth who carried a gun in the prior time-point, even after controlling for a comprehensive set of covariates (Watts, 2019).

### Age Interactions

In general, the analysis in the present study found that the nature of the risk factors for gun carrying largely did not change with age. The only potential exceptions to this were in regard to prior gun carrying and theft and property offending, which may be stronger predictors of gun carrying at younger ages. These results suggests that gun carrying may have more risk factors in adolescence and that gun carrying may be more sporadic or episodic in early young adulthood than in adolescence. However, it is important to consider that the vast majority of the age analyses were not significant, suggesting that the predictors of gun carrying generally did not change across adolescence and the transition to adulthood.

### Correspondence Between Predictors of Gun Carrying and Other Antisocial/Illegal Behaviors

An additional question examined in the present study was whether the risk factors for gun carrying were different than the risk factors for other non-gun offending. From a practical standpoint, it is important to examine the unique predictors of gun carrying to determine the extent to which established intervention and prevention programs for other conduct problems should be tailored to specifically address gun carrying or whether they could be applied broadly. The analysis in the present study found that the factors significantly related to gun carrying were mostly circumscribed to the gun-related risk factors and co-occurring other offending, while a variety of risk factors were related to the non-gun offending outcomes. For example, future orientation, impulse control, peer general (non-gun) offending, parent general (non-gun) offending, and exposure to non-gun violence were all significantly related to theft and property offending and violent and aggressive offending, while none of these factors were significantly associated with gun carrying. As such, the findings in the present study suggest that the unique risk factors for youth gun carrying should be taken into consideration when designing and implementing gun violence prevention and intervention programs. One of the key goals of programs designed to reduce gun carrying should be to reduce young men’s real, implied, and perceived exposure to gun-related events in their social networks and communities.

### Limitations

The results from the present study should be considered in light of the study limitations. For example, the authors examined the research questions with data from a sample of male youth who were recruited into the study after their first contact with the juvenile justice system. As such, results may not generalize to other demographic groups (e.g., clinical, women, community samples). However, it was interesting that many of the observed associations in the present study were consistent with prior published work conducted with a slightly older study of serious adolescent offenders. Nonetheless, future research should examine the research questions with other samples. In addition, the mechanisms linking the significant risk factors to gun carrying were not directly measured, although it was hypothesized that peer gun carrying was significantly related to youth gun carrying because friends modeled, encouraged, or normalized gun carrying (consistent with socialization processes) and that exposure to gun violence was significantly related to gun carrying because youth who were the victims of gun violence were motivated to protect themselves or retaliate. However, it could be that all of these risk factors correlated with youth gun carrying because these are all symptoms of an environment where firearms are ubiquitous and accessible. It is important for future work to examine whether firearm access impacts the associations observed in the present study. Prior studies show that youth who perceive easier access to guns are more likely to carry and use guns than other youth (Gonzales & McNiel, 2020; Hemenway et al., 2011; Keil et al., 2020; Lizotte et al., 2000; Molnar et al., 2004), but none of these studies examined whether perceived gun access is associated with youth gun use in within-individual change models. Unfortunately, perceived gun access was not measured in the present study. In addition, our measures of peer and parent gun carrying and other offending were based on youths’ perceptions of peer and parent behavior and may not perfectly represent their true behavior. One study suggests that youth may overestimate the prevalence of peer gun carrying (Hemenway et al., 2011). Future work should consider including reports directly from peers and parents to determine the extent to which youths’ perceptions match reality. Moreover, our measure of gun violence exposure—being shot or witnessing someone being shot—was a relatively severe measure of gun violence and thus it is unclear whether being threatened or witnessing someone threatened with a gun, but not shot, would be similarly related to youth gun carrying. Furthermore, it is possible that interactions among the risk factors were significantly related to gun carrying. Consistent with this idea, one prior study found that the impact of peer gun carrying was stronger for youth with low callous and unemotional traits (Robertson et al., 2020). Although the present study was not able to include all possible interactions, this is an area for future research. Additionally, the measure of gun carrying did not distinguish between legal and illegal gun carrying. While gun carrying is illegal for minors, some states allow adults to own and carry guns in some capacity. Two of the sites in the present study, Pennsylvania (https://www.psp.pa.gov/firearms-information/Pages/Carrying-Firearms-in-Pennsylvania.aspx) and Louisiana (https://gunlawsuits.org/gun-laws/Louisiana/open-carry/), tend to have more permissive gun laws, while the third site, California, has some of the strictest gun laws in the country (https://oag.ca.gov/sites/all/files/agweb/pdfs/firearms/pdf/cfl2016.pdf; https://oag.ca.gov/firearms/pubfaqs#1). Finally, it is important to keep in mind that almost all of the risk factors and the outcome, gun carrying, were measured at the same time-period. It is hypothesized that the direction of the effect was from the risk factors to gun carrying, but the precise temporal ordering was not tested in the present study.

## Conclusion

Prior studies have identified a handful of potential explanations for gun carrying during adolescence and early young adulthood. For example, studies have suggested that adolescents and young adult are more likely to carry guns if their peers carry guns, if they have been exposed to violence, and if they have low psychosocial maturity. However, no prior study has simultaneously examined several potential risk factors in a dynamic within-individual change model, controlled for youth’s prior gun carrying and other co-occurring offending, included a variety of gun and non-gun related sources of social influence, included both peer and parent factors, and examined whether the magnitude of the predictors change with age. The present study found that the factors with the strongest associations with gun carrying during adolescence and the transition to young adulthood were largely circumscribed to other gun-related events, including peer gun carrying and exposure to gun violence, and co-occurring offending, including theft and property offending and aggressive and violent offending. Similar to communicable diseases, the results support the idea that a gun-related event may produce a ripple or contagion effect (see Green et al., 2017; Towers et al., 2015), which demonstrates that efforts to reduce gun violence should work to prevent the transmission of gun carrying from one person to another within a social network. Physicians, parents, and educators who learn about a young person’s exposure to a gun-related event should screen and refer youth to targeted prevention and intervention programs to prevent future gun carrying among those exposed. Based on the results in the present study, gun violence intervention and prevention programs should be tailored to specifically emphasize the reduction of adolescents’ and young adults’ real and perceived exposure to gun-related events in their communities, in addition to reducing overall levels of criminal recidivism among justice-system involved youth.

## Supplementary information

Supplementary Tables
