# Supplementary material for: A Within-Individual Examination of the Predictors of Gun Carrying During Adolescence and Young Adulthood Among Young Men
Source: J Youth Adolesc. 2021 Jul 16;50(10):1952–69. doi: 10.1007/s10964-021-01464-6 (PMC8417009; doi:10.1007/s10964-021-01464-6)
Supplement: Supplementary file 1 — Supplementary Tables [file 10964_2021_1464_MOESM1_ESM.docx]

Supplemental Table 1

*Model comparison tests between fully adjusted primary model with time-stable effects and models wherein each risk factor was freely estimated*

|  |  |  | Time 2 | Time 3 | Time 4 | Time 5 | Time 6 | Time 7 | Time 8 | Time 9 |
| --- | --- | --- | --- | --- | --- | --- | --- | --- | --- | --- |
|  | X^2^ | *p* | *B* (*p*) | *B* (*p*) | *B* (*p*) | *B* (*p*) | *B* (*p*) | *B* (*p*) | *B* (*p*) | *B* (*p*) |
| Psychosocial Maturity |  |  |  |  |  |  |  |  |  |  |
| Impulse control | 8.20 | .315 |  |  |  |  |  |  |  |  |
| Future orientation | 12.56 | .016 |  |  |  |  |  |  |  |  |
| Behavioral |  |  |  |  |  |  |  |  |  |  |
| Gun carrying | **31.35** | **<.001** | **1.389 (.007)** | **1.937 (<.001)** | **1.005 (.039)** | 0.588 (.290) | **2.045(<.001)** | 1.392 (.074) | 0.308 (.730) | -0.124 (.923) |
| Non-gun theft and property offending | 4.21 | .263 |  |  |  |  |  |  |  |  |
| Non-gun aggressive and violent offending | 0.60 | .998 |  |  |  |  |  |  |  |  |
| Drug dealing | 8.82 | .266 |  |  |  |  |  |  |  |  |
| Social Influence |  |  |  |  |  |  |  |  |  |  |
| Peer gun carrying | 7.02 | .426 |  |  |  |  |  |  |  |  |
| Peer general (non-gun) offending | **22.09** | **.002** | 0.019 (.936) | -0.124 (.568) | -0.022 (.928) | -0.014 (.955) | 0.497 (.126) | **0.827 (.049)** | 6.666 (.325) | -0.156 (.932) |
| Parent gun carrying | 9.58 | .214 |  |  |  |  |  |  |  |  |
| Parent general (non-gun) offending | 11.48 | .119 |  |  |  |  |  |  |  |  |
| Victimization |  |  |  |  |  |  |  |  |  |  |
| Exposure to gun violence | 0.60 | .998 | **1.666 (<.001)** | **1.767 (<.001)** | **2.113 (<.001)** | **1.538 (.001)** | **1.620 (.003)** | **1.419 (.018)** | **1.898 (.001)** | 0.279 (.806) |
| Exposure to general (non-gun) violence | **15.17** | **.003** | 0.561 (.205) | -0.245 (.504) | **1.175 (.019)** | -0.085 (.823) | 0.504 (.250) | 0.811 (.061) | 1.949 (.357) | -0.149 (.818) |

*Notes.*  Model comparison tests were conducted with log likelihood X^2^ tests. Each X^2^ test in the table is the result of a comparison between a model where the parameters are constrained to be equal across time (primary model) to a model where each risk factor (individually) is freely estimated across time. A significant X^2^  test suggests that the freely estimated model may be a significantly better fit to the data than a constrained model. Bold typeface added to emphasize parameters that were significant based on *p* < .05.

Supplemental Table 2

*Interactions between age at time 1 and time-varying risk factors*

|  |  |  |
| --- | --- | --- |
|  | *B* | *p* |
| Psychosocial Maturity |  |  |
| Impulse control | 0.01 | .854 |
| Future orientation | -0.17 | .131 |
| Behavioral |  |  |
| Gun carrying | -0.24 | .116 |
| Non-gun theft and property offending | **-0.26** | **.023** |
| Non-gun aggressive and violent offending | 0.03 | .813 |
| Drug dealing | -0.06 | .739 |
| Social Influence |  |  |
| Peer gun carrying | 0.01 | .812 |
| Peer general (non-gun) offending | -0.14 | .051 |
| Parent gun carrying | -0.39 | .144 |
| Parent general (non-gun) offending | **-0.43** | **.005** |
| Victimization |  |  |
| Exposure to gun violence | -0.19 | .129 |
| Exposure to general (non-gun) violence | -0.00 | .977 |

*Notes.*  Interactions examined in primary model (fixed effects dynamic panel model with all covariates). Bold typeface added to emphasize parameters that were significant based on *p* < .05.

Supplemental Table 3

*Sensitivity analyses: Within-individual associations between risk factors and gun carrying*

| Predictor | *B* | *SE* | *p* |
| --- | --- | --- | --- |
| Psychosocial Maturity |  |  |  |
| Impulse control | 0.11 | 0.13 | .413 |
| Future orientation | -0.10 | 0.17 | .533 |
| Behavioral |  |  |  |
| Prior gun carrying | **1.23** | **0.26** | **<.001** |
| Non-gun theft & property offending (count) | **0.39** | **0.08** | **<.001** |
| Non-gun aggressive & violent offending (count) | **0.38** | **0.11** | **.001** |
| Drug dealing | 0.27 | 0.22 | .204 |
| Social Influence |  |  |  |
| Peer gun carrying | **0.87** | **0.10** | **<.001** |
| Peer general (non-gun) offending | -0.16 | 0.15 | .282 |
| Parent gun carrying | 0.23 | 0.34 | .494 |
| Parent general (non-gun) offending (count) | **0.23** | **0.11** | **.029** |
| Victimization |  |  |  |
| Exposure to gun violence | **1.63** | **0.21** | **<.001** |
| Exposure to general (non-gun) violence | 0.28 | 0.18 | .130 |
| Time-Stable Demographic Factors |  |  |  |
| Race & Ethnicity |  |  |  |
| Black | 0.29 | 0.30 | .329 |
| Hispanic | 0.07 | 0.30 | .829 |
| Other | -0.07 | 0.58 | .905 |
| Parent highest education | 0.07 | 0.04 | .108 |
| IQ proxy | -0.01 | 0.01 | .431 |
| Age at 1st arrest | 0.05 | 0.06 | .461 |

*Notes.* Model was estimated with a binary fixed-effects logistic regression in a structural equation framework with maximum likelihood estimation (dynamic panel models). Missing data were imputed with 25 datasets. All models also controlled for time. All predictor variables were concurrent with the outcome except the time-invariant demographic variables (which were measured at baseline) and the lagged dependent variable (lagged interval = Time – 1). The only difference between this sensitivity model and the primary model is that three variables that were dichotomized in the primary model were left in count form here (e.g. non-gun theft and property offending; non-gun aggressive and violent offending; parent non-gun offending). Bold typeface added to table to emphasize findings that were significant based on *p* < .05.

Supplemental Table 4

*Sensitivity analyses: Within-individual associations between risk factors and gun carrying in original data (non-imputed; N = 497)*

| Predictor | *B* | *SE* | *p* |
| --- | --- | --- | --- |
| Psychosocial Maturity |  |  |  |
| Impulse control | 0.10 | 0.25 | .684 |
| Future orientation | -0.65 | 0.36 | .068 |
| Behavioral |  |  |  |
| Prior gun carrying | 0.56 | 0.40 | .161 |
| Non-gun theft & property offending | 0.86 | 0.57 | .129 |
| Non-gun aggressive & violent offending | 0.43 | 0.55 | .430 |
| Drug dealing | **1.01** | **0.35** | **.004** |
| Social Influence |  |  |  |
| Peer gun carrying | **1.01** | **0.38** | **.007** |
| Peer general (non-gun) offending | -0.06 | 0.29 | .825 |
| Parent gun carrying | 0.15 | 0.82 | .856 |
| Parent general (non-gun) offending | 0.42 | 0.49 | .391 |
| Victimization |  |  |  |
| Exposure to gun violence | **1.91** | **0.38** | **<.001** |
| Exposure to general (non-gun) violence | 0.56 | 0.40 | .165 |
| Time-Stable Demographic Factors |  |  |  |
| Race & Ethnicity |  |  |  |
| Black | -0.13 | 0.78 | .868 |
| Hispanic | -0.55 | 0.85 | .521 |
| Other | 0.17 | 2.17 | .939 |
| Parent highest education | -0.05 | 0.10 | .580 |
| IQ proxy | -0.01 | 0.03 | .754 |
| Age | 0.07 | 0.23 | .756 |

*Notes.* Model was estimated with a binary fixed-effects logistic regression in a structural equation framework with maximum likelihood estimation (dynamic panel models) with original raw data (no imputed data). All models also controlled for time. All predictor variables were concurrent with the outcome except the time-invariant demographic variables (which were measured at baseline) and the lagged dependent variable (lagged interval = Time – 1). Bold typeface added to table to emphasize findings that were significant based on *p* < .05.
